# Where is the zero of Tardieu for proximal trans-joint lower limb muscles? The relevance for the estimation of muscle shortening and weakness

**DOI:** 10.3389/fneur.2023.1108535

**Published:** 2023-04-28

**Authors:** Maud Pradines, Tymothée Poitou, Ota Gál, Martina Hoskovcová, Nicolas Bayle, Marjolaine Baude, Jean-Michel Gracies

**Affiliations:** ^1^UR 7377 BIOingénierie Tissus Neuroplasticité (BIOTN), Faculté de Santé, Université Paris-Est Créteil, Créteil, France; ^2^Service de Rééducation Neurolocomotrice, Hôpitaux Universitaires Henri Mondor, Créteil, France; ^3^Department of Neurology, Charles University, Prague, Czechia

**Keywords:** modified Tardieu scale, five step assessment, coefficient of shortening, hemiparesis, zero of Tardieu, quantified assessment

## Introduction

In the quoted retrospective study (1), we used a quantified clinical measurement, the five-step assessment (an expansion of the Tardieu scale), that aims to quantify each of the neurological and the muscle disorders and distinguish them from one another as much as is clinically possible (2). We thus estimated muscle shortening and command impairment against key muscle groups of the lower and upper limbs, which led us to suggest a deleterious impact of structural muscle changes on the quality of the motor command in chronic stroke-induced hemiparesis.

## Brief history

Tardieu developed a clinical method to discriminate between various forms of passive resistance from antagonist muscle groups (3). Using his methodological principles, we created the *Tardieu scale*, in which the first parameter X_V1_ reflects muscle extensibility mostly, using slow and strong passive stretching movement of the evaluated muscle (4, 5). The quantification of spasticity was achieved through the comparison of X_V1_ with the angle of catch, X_V3_, obtained during a passive phasic stretch performed at high velocity and through the measurement of the grade of spasticity Y, reflecting the type of muscle reaction (4, 5). According to Tardieu's methods, X_V1_ and X_V3_ were measured from a theoretical “zero” that was defined for each muscle as its *theoretical position of minimal stretch* (3). As the scale was being developed, the practicality of assessment—keeping in mind the need for easy dissemination of the scale—led us to adjust the *theoretical* zero from Tardieu's initial proposal for a few specific muscles only, for example, the zero for shoulder extensors was selected at the shoulder in neutral (0° flexion or extension) instead of 50° or 90° shoulder extension (4–6).

We later expanded the X_V1_ and X_V3_ of the Tardieu Scale into a five-step assessment, where a functional step (Step 1, guiding the following) and another two technical steps were added, X_A_ and X_A15_ (7). The latter two parameters correspond to the maximal active range of motion against passive and active antagonist resistances in a single effort (X_A_, Step 4) and after 15 s of repeated maximal amplitude movements (X_A15_, Step 5) (7).

In an attempt to help the clinician to better distinguish between the muscle and the neural disorders and to provide guidance toward relevant therapeutical strategies, we defined specific coefficients of impairment from X_V1_, X_V3_, and X_A_ as follows (2):

Coefficient of shortening: C_SH_ = (X_N_ – X_V1_)/X_N_, where X_N_ is the normally expected maximal passive joint amplitude for each tested muscle,
Coefficient of spasticity: C_SP_ = (X_V1_ – X_V3_)/X_V1_
Coefficient of weakness: C_W_ = (X_V1_ – X_A_) / X_V1_.

Using this calculation methodology, the definition of the zero is of importance as it will directly cause an impact on the value of all parameters and thus on each of the aforementioned coefficients. For most one-joint muscles except plantar flexors, X_N_ usually ends up at 180° or 150°, which makes calculations relatively homogeneous across muscles, and these angular values typically correspond to functional muscle excursions in real-life movements.

## Discussion

### Incidence of the zero of Tardieu for large trans-joint lower limb muscles

Overall, two proximal trans-joint muscles in the lower limb, rectus femoris (RF) and hamstrings (HS), still cause a dilemma as to where to position the zero. When using the zero based on the *theoretical* position of minimal stretch (hip flexed at 90° and knee extended for RF; hip in neutral and knee flexed at 180° for HS), X_N_ ends up far >180°: 240° for RF (courses covering 150° knee flexion + 90° hip extension from the zero) and 270° for HS (courses covering 180° knee extension + 90° hip flexion from the zero). These large X_N_ values bear important consequences as they lead to potential underestimation of the coefficients of shortening (X_N_ – X_V1_)/X_N_ and of weakness (X_V1_ – X_A_)/X_V1_, particularly as a significant part of this theoretical 240 ° or 270° X_N_ range is far from functional for these muscles.

**Figure 1:**
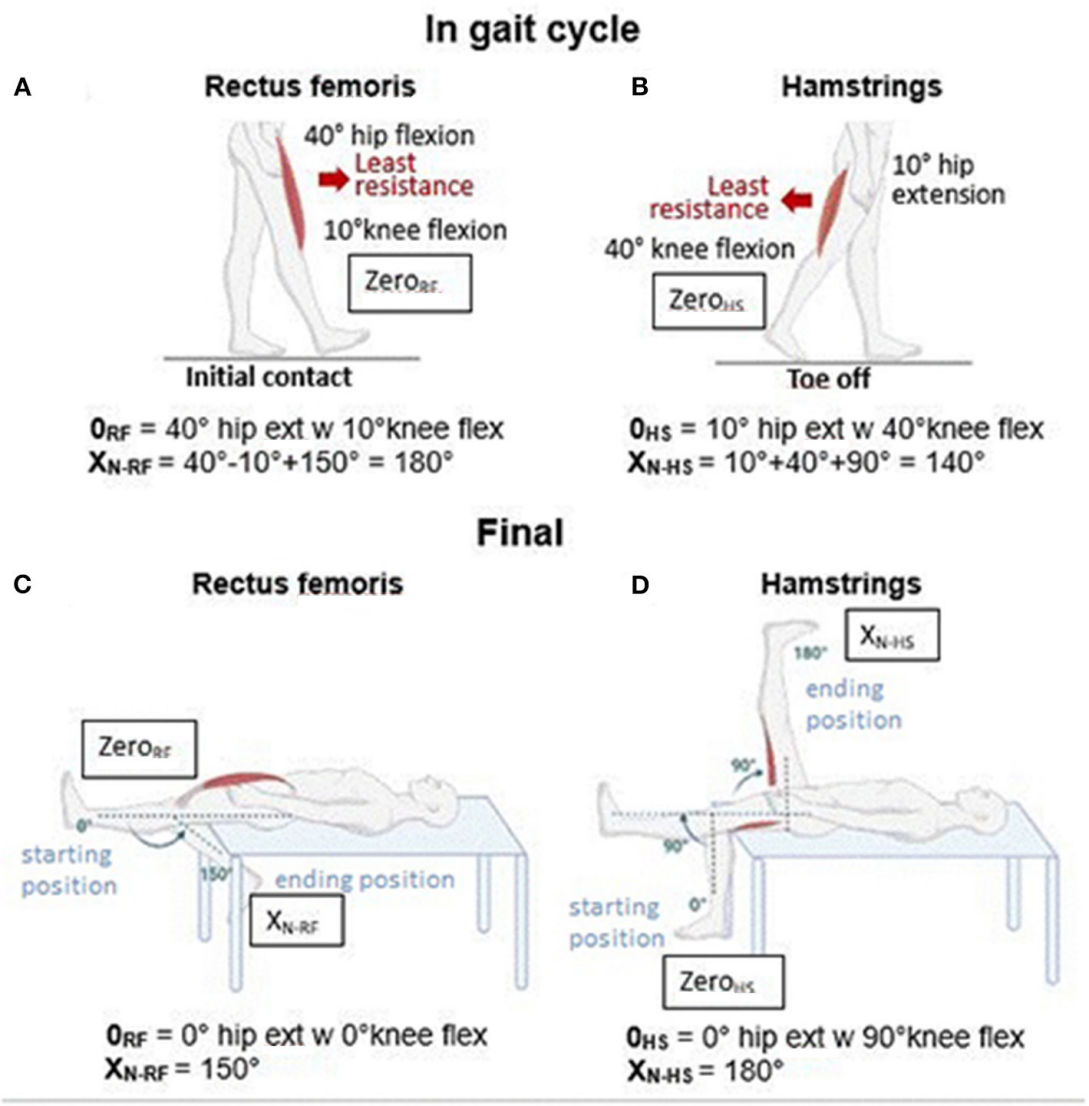
“Zero” and X_N_ for rectus femoris and hamstrings. **(A)** Zero position of the rectus femoris, noted as 0_RF_ in Figure, is exemplified here as based on the position of its least antagonist resistance during the gait cycle. **(B)** Zero position of hamstrings, noted as 0_HS_ in Figure, is exemplified here as based on the position of its least antagonist resistance during the gait cycle. **(C)** Final zero and X_N_ of rectus femoris. **(D)** Final zero and X_N_ of hamstrings. w = with.

Under these considerations, we have revisited the specific definition of the *zero of Tardieu* for these two muscles in an attempt to bring it closer to a “functional zero” (Figures 1A, B) or, in other words, to a position of a minimal stretch of the muscle *while engaged in actual functional activities*. The primary functional activity involving the lower limbs is gait. For these trans-joint muscles, we might aim for higher functional relevance in positioning their “zeros” at their *points of least antagonist resistances (due to shortening and/or spastic cocontraction) during a standard gait cycle*. In subjects with spastic paresis, the least antagonist resistance from the rectus femoris might occur at late swing (heel on) when the hip is flexed to approximately 40° and the knee is flexed to approximately 10° (Figure 1A) (8). The X_N_ would then be the difference in rectus femoris excursion between that position and the maximally stretched position of the muscle (hip at 0° extension and knee at 150° flexion), i.e., 180°.

As for the hamstrings, the point of least antagonist resistance during a standard gait cycle comes at the end of the stance (toe off) when the hip is extended ~10° and the knee is flexed to ~40° (Figure 1B) (8). The X_N_ would then be the difference in excursion of the hamstrings between that position and the maximally stretched position of the muscle (hip at 90° flexion and knee at 0° flexion), i.e., 140°.

However, these “functional” ranges will vary when considering other functional activities such as running, climbing up, or walking down the stairs. Therefore, to combine improved functional relevance, practicality, and an X_N_ as close as possible to 180°, we propose the following arbitrary zero for these two muscles:

- For RF, the “neutral” standing position, hip at 0° extension, and knee at 0° flexion, which brings X_N_ to 150° (Figure 1C).
- For HS, the position hip extension at 0° and knee flexed at 90°, which brings X_N_ to exactly 180° (Figure 1D).

The clinical method to practically examine the rectus femoris and the hamstring using the five-step assessment has been published earlier (9). As a reminder, maximal clinical extensibility of the rectus femoris X_V1 − RF_ is evaluated as follows: the subject lies supine at the end of the table, with the paretic leg dangling over the table with the knee flexed. The clinician flexes the knee very slowly, maintaining the hip in extension.

As for the measurement of hamstrings extensibility X_V1 − HS_, the subject is supine with the lower limb lying straight on the table. The clinician slowly flexes the hip, keeping the knee extended throughout the movement. For the active movement against the passive and active resistances of the rectus femoris (X_A − RF_) and hamstrings (X_A − HS_), the same movement as described above will be attempted by the patient himself. When using the scale, it should be noted that the labels *gastrocnemius* and *rectus femoris* stand for the muscle groups, “*gastrocnemius* + *soleus* + *other plantar flexors”* and for “*rectus femoris* + *vastus medialis*+ *vastus lateralis* + *vastus intermedius,”* respectively. The reason for this simplification is that the angles X_V1_, X_V3_, and X_A_ with these muscle groups are systematically lower than those against the resistance of single joint muscles *soleus* (knee flexed) and *vastus muscles* (hip flexed) alone, respectively, which points to the primary responsibility of the trans-joint muscles in these muscle group angles (1, 9, 10).

### Reconsideration of the assessment of shortening in proximal trans-joint lower limb muscles and the weakness of command against their resistance, using the newly defined zeros

Using these new zeros in the above-cited study (1), the coefficients of shortening and the weakness of the rectus femoris were obtained as follows: C_SH − RF_ = 0.21 ± 0.11, C_W − RF_ = 0.28 ± 0.18 (as against C_SH − RF_ = 0.13 ± 0.07 and C_W − RF_ = 0.15 ± 0.09 in the original version). For the hamstrings, the new coefficients were C_SH − HS_ = 0.12 ± 0.10, C_W − HS_ = 0.26 ± 0.17 (as against C_SH − HS_ = 0.06 ± 0.05 and C_W − HS_ = 0.12 ± 0.08 in the original version).

These data led us to suggest that rectus femoris shortening, in particular, might have been markedly underestimated in previous studies using the five-step assessment. Using this modified calculation modality, the rectus femoris ends up getting estimated as the most shortened muscle of the lower limb, beyond gastrocnemius shortening (C_SH − GN_, 17%). The present results thus tend to corroborate previous findings, demonstrating the overwhelming predominance of rectus femoris functional impact on gait impairment (10). As for motor command against the resistance from the rectus femoris, the new value of C_W − RF_ ends up at 0.28 ± 0.18, which has been previously identified as the highest coefficient of weakness in the lower limb, [1] beyond that against gastrocnemius resistance as well (C_WK − GN_, 21%).

These changes in clinical estimates are of great significance, as rectus femoris shortening and/or cocontraction lead, while walking, to what has been termed an *anterior pattern*, which is a frequent pattern seen after ischemic stroke in particular (2). On the paretic side, passive and active resistances from this muscle will gradually increase throughout the stance, leading to insufficient knee flexion in pre-swing and thus an inappropriate preparation of swing, reduced foot clearance, and further knee flexion during the swing and decreased cadence.

On the other side of the thigh, the impairment of motor command against the resistance from hamstrings muscles is also drastically re-evaluated (C_WK − HS_, 26%) while using the new X_N − HS_, ending just below the C_WK − RF_. As for its functional implications, hamstring spastic cocontraction—and/or shortening—leads to a *posterior pattern* during gait, with a lack of knee re-extension (on the paretic side) at the end of the swing phase. This will bring about a decrease in paretic step length and an increase in cadence. Posterior patterns are common after spinal cord lesions and some atypical strokes (2).

Therefore, from a therapeutical standpoint, both muscle shortening and active command should be treated—particularly in the rectus femoris—through specific techniques (cf. guided self-rehabilitation contract) that will target plasticity in the muscle and the nervous system, respectively.

## Author contributions

All authors listed have made a substantial, direct, and intellectual contribution to the work and approved it for publication.
